# Hybrid classical-quantum machine learning based on dissipative two-qubit channels

**DOI:** 10.1038/s41598-022-24346-8

**Published:** 2022-11-28

**Authors:** E. Ghasemian, M. K. Tavassoly

**Affiliations:** Laser and Optics Group, Faculty of Physics, Yazd University, Yazd, Iran

**Keywords:** Optics and photonics, Physics

## Abstract

Although the environmental effects, i.e., dissipation and decoherence seem to be the strongest adversaries in the quantum information realm, here, we address how dissipation can be harnessed for quantum state preparation and universal quantum computation. In this line, we propose a realistic scheme for hybrid classical-quantum neural networks based on dissipative two-qubit channels. In particular, we design a variational quantum circuit consisting of a set of universal quantum gates. We encode classical information in the initial states of a two-qubit system interacting with a global environment. This composite system plays the role of a dissipative quantum channel (DQC). A pooling layer concatenates the output states of the DQCs resulting in the outcome of the circuit. Both the DCQs and the pooling layer provide superposition and entanglement which are the key ingredients of any universal quantum computation protocol. Finally, we investigate the capability and adaptability of this model by doing some machine learning tasks. It is reasonable to postulate that a quantum computer based on DQCs may outperform a classical computer because, in contrast to the latter, the former is capable of producing atypical patterns through non-classical phenomena.

## Introduction

The major challenge in the implementation of a quantum computer lies in the difficulty to maintain coherence to simulate unitary dynamics. Naturally, real physical systems interact with their environments and undergo the effects of dissipation (loss in the population of quantum states) and decoherence (destruction of quantum state correlations)^1^. Dissipation and decoherence are the strongest adversaries in quantum information science that destroy and wash out the quantum resources that improve the power of quantum computation, cryptography and quantum simulation^2–4^. Although this issue seems to be true for many physical situations, nevertheless it has been shown that dissipation can also behave exactly in the opposite manner, i.e., it may facile universal quantum computation. Indeed, dissipation can be exploited to drive a system to a desired steady state where the outcome of the computation is encoded^5,6^. The idea of dissipative quantum computing is of fundamental interest for quantum neural network (QNN) research, since it facilitates quantum computation based on the dynamic attractors and steady states. The authors in^5^ showed that dissipative quantum computation is robust in the sense that, the system is driven towards its steady state independent of the initial state and hence of eventual perturbations along the way.

On the other hand, quantum computing has demonstrated its superiority to deal with the problems intractable on classical computers, such as factorization of large numbers and searching in an unstructured database^3,7–9^. Meanwhile, quantum computation with large circuit depth encounters essential challenges due to the noise associated with the quantum gates and lack of quantum error correction on the noisy intermediate-scale quantum devices^10^. Hence, it would be of fundamental interest to develop quantum algorithms that are resilient to noise with moderate circuit depth. Nowadays, variational quantum algorithms have rapidly been developed in many fields^11^. In particular, quantum machine learning based on variational quantum circuits demonstrates great potential in surpassing the performance of classical machine learning models^12–14^. One of the major advantages of variational quantum models compared to their classical counterparts is the drastic reduction in the number of model’s parameters. Hence, variational quantum models potentially mitigate the overfitting problems associated with classical machine learning. Moreover, they may learn faster or achieve higher testing accuracies compared to their classical counterparts under certain conditions^15^. A modern quantum machine learning architecture typically includes both classical and quantum parts wherein the variational quantum model plays a crucial role as the quantum component with the circuit parameters updated via a classical computer^16^.

In this paper, we propose a hybrid classical-quantum framework for machine learning based on dissipative quantum channels (DQCs). Indeed, we exploit the dissipation to prepare the steady state of a two-qubit system which depends on its initial state. Such a system can be used to establish dissipative quantum computation and quantum information processing. We encode the classical information into the initial states of DQCs that constitute a variational quantum circuit. We show how such a dissipative hybrid classical-quantum model can be used for universal quantum computation. Unlike some quantum machine learning schemes that require a pre-trained process, our proposed architecture is trained as a whole. We demonstrate how to reproduce the classical neural networks (CNNs) with a quantum circuit, making the scheme highly adaptable, particularly, for feed forward NNs. Finally, we apply the scheme for solving binary classifcation probelem, however, it can be used for dealing with more difficult tasks such as ternary classification tasks and in gereral multi-class problems.

This paper is organized as follows. At first, we introduce our strategy for the implementation of dissipative hybrid classical-quantum NNs. Then, we study the dynamics of a two-qubit system under the influence of a global environment. In the continuation, we discuss how such a system can be used for the modeling of dissipative NNs and universal quantum computing. Finally, we show the capability and adaptability of our hybrid model by solving some classification problems.

## Machine learning via DQC

Machine learning is a developing branch of computational algorithms that are introduced to emulate human intelligence by learning from the surrounding environment. Machine learning, as a subfield of artificial intelligence, enables systems to learn and improve from experience without being explicitly programmed^17^. The ability of machine learning algorithms to learn from the current context and generalize into unseen tasks would allow improvements in diverse fields ranging from pattern recognition, computer vision, spacecraft engineering, finance, entertainment, and computational biology to biomedical and medical applications^18–21^.

A machine learning algorithm is a computational process that uses input data to extract a particular outcome or perform a desired task without being literally programmed, i.e., “hard coded”. Such computational algorithms are known as “soft coded” in the sense that they automatically alter or adapt their architecture through repetition (i.e., experience) so that they become better and better to deal with the desired tasks. The adaptation process is called training, in which samples of input data are provided along with desired outcomes^17^.

A machine learning algorithm can adapt itself in response to the training process. This training is the “learning” part of machine learning. The input data can be selected and weighted to provide the most decisive outcomes. The algorithm can possess variable numerical parameters that are adjusted through iterative optimization. Also, it can provide probability distributions from the input data and use them to predict outcomes. A detailed introduction to machine learning and its applications can be found in the literature^17,22^.

“Quantum machine learning” refers to the integration of quantum algorithms within machine learning programs^13,23^. Indeed, quantum machine learning is a subdiscipline of quantum information processing research, that intends to develop quantum algorithms that learn from data in order to improve traditional methods in machine learning.

Currently, one of the preferred tools for quantum machine learning algorithms are the variational (or parametrized) circuits that allow training the quantum devices in the same way as a CNN. Besides, they are able to deal with some machine learning problems that cannot be solved by classical computers in an efficient way^24^. Nowadays, the term QNN is increasingly put forward in the quantum machine learning context. It refers to parametrized quantum and hybrid algorithms that can be optimized or trained by a classical processor^25,26^. QNNs include models, systems or devices that combine features of quantum theory with the properties of NNs. Since two-level systems (qubits) constitute the basic unit of quantum computing, so they can give an interconnection between NN models and quantum theory. Consequently, quantum neurons as the building blocks of QNN may be constructed from two-level quantum systems. Accordingly, the majority of QNN models have been proposed based on the idea of the qubit neuron (quron)^1^.

Furthermore, the idea of open QNN has been put forward based on the theory of open quantum systems^27,28^ and the promising field of dissipative quantum computing^5^. Open QNN models exploit dissipation in order to investigate dynamical properties similar to NNs^1^.

The so-called dissipative quantum computing model is established based on the theory of open quantum systems wherein quantum systems interact with a large environment. The total system consisting of the principal system plus its environment still undergoes a unitary evolution, but the principal system alone propagates nonunitarly and experiences decoherence^5^.

Hence, we are motivated to propose a hybrid classical-quantum machine learning model based on a dissipative two-qubit system under the influence of a global environment. In particular, we design a variational quantum circuit that consists of some DQCs and modulators. One can treat a QNN as a black box and control a set of free parameters corresponding to the designed quantum circuit and train the network. Training could then be performed using numerical techniques, i.e., optimizing the circuit parameters with respect to a cost function.

Generally, quantum computing consists of the preparation, evolution and measurement of qubits. In dissipative quantum computing, the qubits are not only manipulated by unitary quantum gates, but also interact with their environment^1^.

Let us focus on the role of the DQC in our quantum machine learning approach. A two-qubit system under the influence of a global environment, namely a DQC, can be viewed as a quantum gate that can perform different tasks. Firstly, it can prepare the steady state for encoding classical data via the dissipation process. Secondly, it can provide a quantum superposition of the input signals of a single-neuron network. We discuss these features in the following. Besides, a global dissipative environment can establish stationary entanglement. Interestingly, this happens even without any direct interaction between the two qubits^29–33^. Briefly, we can state that our dissipative model is established based on the fact that dissipation can be exploited to drive a system to a desired steady state, where the outcome of the computation is encoded. The authors in^5^ showed how dissipation can be used to engineer a large variety of strongly correlated states in steady state, including all stabilizer codes, matrix product states and their generalization to higher dimensions. Also, it should be noted that, the unique quantum mechanical features such as superposition and entanglement can be used to solve hard computational tasks with quantum computers significantly faster compared to the best known classical algorithms^8,34^. Hence, it would be fruitful to explore the role of fundamental quantum physics concepts such as superposition, entanglement and dissipation in quantum machine learning more deeply.

## Method

**Figure 1:**
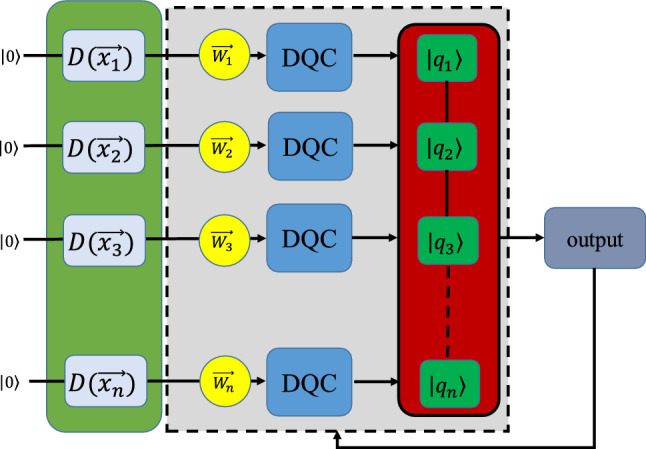
A quantum circuit is composed of an input layer (for encoding data), an intermediate layer (for processing data), and an output layer (for providing output signal). The classical data can be encoded into quantum states by the action of the displacement operator on the vacuum state ${\mathcal {D}}(x)\vert 0\rangle =\vert x\rangle$. Indeed, the *i*-th signal $\vec {x}_i=(x_1,x_2,x_3)$ is encoded in the initial state of the *i*-th DQC. The processing unit constitutes some quantum operations such as adjustable electro-optical modulators, DQCs, and a pooling layer. The output signal of the intermediate layer is fed into the output layer. This quantum circuit serves as a NN.

Here, we develop a variational QNN model based on DQCs to be implemented as a supervised classifier. In this line, we design a quantum circuit consisting of an input layer, an intermediate layer (including some electro-optical modulators and DQCs, and SUM gates), and an output layer as schematically depicted in Fig. 1.

At first, the data are encoded into the quantum state with the help of the displacement operations followed by some electro-optical modulators and then enter their corresponding DQCs to be entangled. The output signals of the DQCs pass through a pooling layer including a number of SUM gates to be concatenated, resulting in a superposition of the initial states of DQCs. Finally, the output signal of the NN may be obtained by tracing out the unwanted modes or measurement processes.

Indeed, the data are encoded in the initial states of *n* two-qubits coupled to their global environments. It should be noted that the coupling to the global environments, which is dissipative in nature, drives the two-qubit systems to their steady states. Therefore, the inputs and outputs of DQCs are the initial and final states of the two-qubit systems.

As in the case of the traditional NN, a pooling layer is required to concatenate the input of the network. There exist several pooling approaches in machine learning contexts. For instance, the pooling layers may be inserted between convolutional layers for feature abstraction and dimension reduction. Also, the progressive reduction of the number of qubits in hierarchical QNNs is analogous to the pooling operation in convolutional NNs^35^. The pooling operation can be presented by a controlled unitary transformation or by quantum nondemolition techniques^36^. Here, we consider a pooling layer to concatenate the outcomes of DQCs and provide a weighted sum from the input signals using a quantum nondemolition sum gate analogous to the controlled-NOT (C-NOT) gate for qubits but without the cyclic condition. This so-called sum gate provides a two-mode entangling gate for universal quantum computation in the regime of continuous variables^36^. Finally, the outcome of the circuit which can be obtained by the homodyne measurement is used for training the NN and controlling the circuit’s parameters. Indeed, a classical computer can be used to compute the new set of parameters, therefore, the parameterized quantum circuit is updated accordingly for the subsequent round.

Using the designed circuit and the known quantum concepts, we show how such unwanted environmental effects such as dissipation can be harnessed for the implementation of universal quantum computation via dissipative two-qubit systems.

## Dissipative two-qubits

The theory of open quantum systems plays an important role in many different applications in the field of dynamical quantum systems^27,28^. Indeed, it is often not possible to isolate the systems or to control the environmental degrees of freedom. Hence, open quantum system techniques are vital for the study of real quantum systems. Indeed, closed quantum systems are just an idealization of real systems. In practical problems, the interaction of the system of interest with the environment cannot be avoided, and one requires an approach in which the environment can be effectively removed from the equations of motion. In other words, in open quantum theory, the goal is to infer the equations of motions of the reduced systems from the equation of motion of the total system. A particular and interesting case is a system connected to several baths, modeled by a Markovian interaction. In this case, the most general quantum dynamics is generated by the Lindblad equation (also called Gorini-Kossakowski-Sudarshan-Lindblad equation)^37^

1 $$\begin{aligned} \frac{d}{dt}\rho _{\mathrm{{s}}}(t)= & {} -i [{\hat{H}},\rho _{\mathrm{{s}}}(t)]+{\mathcal {L}}\rho _{\mathrm{{s}}}(t), \end{aligned}$$

where $\rho _{\mathrm{{s}}}(t)$ and ${\hat{H}}$ are the density operator and Hamiltonian of the system, respectively. Also, ${\mathcal {L}}$ is a linear (super) operator acting on the space of linear operators. Commonly, the Hamiltonian ${\hat{H}}$ represents the unitary dynamics of the system in the absence of the environment, while the operator ${\mathcal {L}}$ describes the non-unitary part of the evolution of the system. Hence, the latter is often called the dissipator environment. The general form of the so-called Lindblad master equation reads as^38^

2 $$\begin{aligned} \frac{d}{dt}\rho _{\mathrm{{s}}}(t)=-i \left[ {\hat{H}},\rho _{\mathrm{{s}}}(t) \right] - \sum _{k=1}^{d^2-1} \gamma _k \left( A^\dagger _kA_k \rho _{\mathrm{{s}}}(t) +\rho _{\mathrm{{s}}}(t) A^\dagger _kA_k-2A_k \rho _{\mathrm{{s}}}(t) A^\dagger _k \right) , \end{aligned}$$

where $\rho _{\mathrm{{s}}}$ is the reduced density matrix characterized by the system which can be gained by taking the partial trace over the degrees of freedom of the environment on the combined (total) system, i.e, $\rho _{\mathrm{{s}}}= \mathrm{Tr_E \rho _{\textrm{T}}}$ where $\rho _{\textrm{T}}$ is defined as the total density operator. Besides, $A_k$s denote the Lindblad operators weighted with the so-called decoherence parameters $\gamma _k$ and *d* is the dimension of Hilbert space. An important phenomenon regarding open quantum systems is the dissipation of energy into the environment. Quite generally, one can deal with the environment as a distribution of the uncorrelated thermal equilibrium mixture of states. The dissipative dynamics of a two-qubit system interacting with its environment is described by a master equation of Lindblad form. The Lindblad operators are described in terms of the lowering and raising operators $\sigma _i$, $\sigma _i^\dagger$, respectively, where $\sigma _i= \vert g\rangle _i\langle e\vert _i$ and $\vert g\rangle$ and $\vert e\rangle$ denote the ground and excited states of the *i*th qubit. As is clear, these operators act locally on the two qubits so that they can be used to describe the dynamics of local dissipation. The continuous miniaturization of physical devices makes them closely spaced, therefore, a two-qubit system also experiences the global dissipation in such a way that the two qubits can interact with a common thermal environment, simultaneously. In order to describe the global dissipation, additional Lindblad operators can be defined in terms of collective operators $\sigma _1+\sigma _2$ and $\sigma _1^\dagger +\sigma _2^\dagger$. Thus, the following master equation governs the dynamics of the density operator $\rho$ of a two-qubit system under the influences of the global and local environments

3 $$\begin{aligned} \frac{d}{dt}\rho (t)= & {} \gamma \sum _{k=1}^{2} \big [2 {\mathcal {A}}_k \rho (t) {{\mathcal {A}}_k}^\dagger - {\mathcal {A}}_k {{\mathcal {A}}_k}^\dagger \rho (t)-{{\mathcal {A}}_k}^\dagger {\mathcal {A}}_k \rho (t) \big ] \nonumber \\{} & {} + (1-\gamma )\sum _{k=3}^{6} \big [2 {\mathcal {A}}_k \rho (t) {{\mathcal {A}}_k}^\dagger - {\mathcal {A}}_k {{\mathcal {A}}_k}^\dagger \rho (t) -{{\mathcal {A}}_k}^\dagger {\mathcal {A}}_k \rho (t) \big ], \end{aligned}$$

where the Lindblad operators are given as

4 $$\begin{aligned} {\mathcal {A}}_1= & {} \sqrt{{\bar{n}}_g+1} \left( \sigma _1+\sigma _2 \right) , \qquad {\mathcal {A}}_2=\sqrt{{\bar{n}}_g}(\sigma _1^\dagger +\sigma _2^\dagger ), \nonumber \\ {\mathcal {A}}_3= & {} \sqrt{{\bar{n}}_l+1} \sigma _1, \qquad {\mathcal {A}}_4= \sqrt{{\bar{n}}_l+1} \sigma _2, \nonumber \\ {\mathcal {A}}_5= & {} \sqrt{{\bar{n}}_l} \sigma _1^\dagger , \qquad {\mathcal {A}}_6= \sqrt{{\bar{n}}_l} \sigma _2^\dagger , \end{aligned}$$

where ${\bar{n}}_g$ and ${\bar{n}}_l$ denote the number of thermal excitations in the global and local environments, respectively. It should be noted that the parameter $\gamma \in [0,1]$ describes the interplay between purely local dissipation ($\gamma =0$) and purely global dissipation ($\gamma =1$).

The global environment can supply a kind of interaction in composite systems that establishes entanglement. Dissipation plays a remarkable role in the open quantum system context because it allows the stabilization of the quantum resources and engineering a large variety of strongly correlated states in the steady state^30,39^. Hence, it provides some key advantages over unitary quantum-state manipulation^40^. Both theoretically^41^ and experimentally^42^, it has been shown that a global dissipative environment can establish stationary entanglement. This notion is a key concept in quantum information and quantum computation sciences. Surprisingly, this may happen even without any direct interaction among subsystems^43^. The simplest model where such a situation can be realized is the case in which two qubits dissipating into a common environment. Here, we are interested in following the dynamics of a two-qubit system under the influence of a global environment. Hence, we set $\gamma =1$ and remove the subscribe of ${\bar{n}}_g$ for the sake of simplicity. Therefore, the dynamics of the two-qubit system will be governed by the following master equation

5 $$\begin{aligned} \frac{d}{dt}\rho (t)= & {} {\mathcal {L}} \rho (t) \nonumber \\= & {} {\bar{n}} \left[ 2 (\sigma _1+\sigma _2) \rho (\sigma _1+\sigma _2)^\dag - (\sigma _1+\sigma _2)^\dag (\sigma _1+\sigma _2)\rho -\rho (\sigma _1+\sigma _2)^\dag (\sigma _1+\sigma _2) \right] \nonumber \\{} & {} +({\bar{n}}+1) \left[ 2(\sigma _1+\sigma _2)^\dag \rho (\sigma _1+\sigma _2)- (\sigma _1+\sigma _2) (\sigma _1+\sigma _2)^\dag \rho -\rho (\sigma _1+\sigma _2) (\sigma _1+\sigma _2)^\dag \right] , \end{aligned}$$

To study the dynamics of a two-qubit system, we can formally expand its density operator in the basis,

$$\begin{aligned} \{|1\rangle := |e_1\rangle |e_2\rangle ;  |2\rangle := |e_1\rangle |g_2\rangle ; |3\rangle := |g_1\rangle |e_2\rangle ; |4\rangle := |g_1\rangle |g_2\rangle \}, \end{aligned}$$

so we arrive at

6 $$\begin{aligned} \rho (t) = \sum _{j,k=1}^4 \rho _{j,k}(t) |j\rangle \langle k|, \end{aligned}$$

where $\rho _{j,k}(t)$ are unknown time-dependent coefficients. Upon the insertion of (6) into (5), the dynamical evolution of the density operator can be described by a set of linear differential equations for the unknown coefficients $\rho _{j,k}(t)$ that can be compactly expressed as

7 $$\begin{aligned} \dot{{\textbf{v}}}(t) = M{{\textbf{v}}}(t), \end{aligned}$$

where

$$\begin{aligned} {{\textbf{v}}}(t) = \left( \rho _{11}(t), \rho _{12}(t), \ldots , \rho _{43}(t), \rho _{44}(t)\right) ^\top , \end{aligned}$$

and *M* is a $16\times 16$ matrix of constant coefficients. Since the general time-dependent solution of this master equation is too cumbersome and as we are interested in the steady state solution, hence its time-dependent solution is not presented.

### Stationary states of the two-qubit system passing through a DQC

Let us start with the following initial state for the system

8 $$\begin{aligned} \rho (0)= \begin{bmatrix} \rho _{11} &{}\quad \rho _{12} &{}\quad \rho _{13} &{}\quad \rho _{14}\\ \rho _{21} &{}\quad \rho _{22} &{}\quad \rho _{23} &{}\quad \rho _{24}\\ \rho _{31} &{}\quad \rho _{32} &{}\quad \rho _{33} &{}\quad \rho _{34}\\ \rho _{41} &{}\quad \rho _{42} &{}\quad \rho _{43} &{}\quad \rho _{44}\\ \end{bmatrix}, \end{aligned}$$

where we set $\rho _{ij}(0)=\rho _{ij}$ and find the solution of the master Eq. (5). As mentioned, the general analytical solution of this master equation is too cumbersome. However, for ${\bar{n}}=0$, the analytical solution can be obtained as below

9 $$\begin{aligned} \rho (t)= & {} \begin{bmatrix} \rho _{11}(t) &{}\quad \rho _{12}(t) &{}\quad \rho _{13}(t) &{}\quad \rho _{14}(t)\\ \rho _{21}(t) &{}\quad \rho _{22}(t) &{}\quad \rho _{23}(t) &{}\quad \rho _{24}(t)\\ \rho _{31}(t) &{}\quad \rho _{32}(t) &{}\quad \rho _{33}(t) &{}\quad \rho _{34}(t)\\ \rho _{41}(t) &{}\quad \rho _{42}(t) &{}\quad \rho _{43}(t) &{}\quad \rho _{44}(t)\\ \end{bmatrix}, \end{aligned}$$

10 $$\begin{aligned} \rho _{11}(t)= & {} \rho _{11} e^{-4t}, \nonumber \\ \rho _{12}(t)= & {} \rho _{21}^*(t)=\frac{1}{2}(\rho _{12}+\rho _{13})e^{-4t}+\frac{1}{2}(\rho _{12}-\rho _{13})e^{-2t}, \nonumber \\ \rho _{13}(t)= & {} \rho _{31}^*(t)=\frac{1}{2}(\rho _{12}+\rho _{13})e^{-4t}-\frac{1}{2}(\rho _{12}-\rho _{13})e^{-2t} \nonumber \\ \rho _{14}(t)= & {} \rho _{41}^*(t)=\rho _{14} e^{-2t}, \nonumber \\ \rho _{22}(t)= & {} \rho _{33}(t)= \left(\frac{3}{2}+2t\right) \rho _{11} e^{-4t}+\frac{1}{8}(-12\rho _{11}+2\rho _{22}+2\rho _{23}+2\rho _{32})e^{-4t} -\frac{1}{2}(-\rho _{22}+\rho _{33})e^{-2t}\nonumber \\{} & {} +\frac{1}{4}(\rho _{22}+\rho _{33}-\rho _{23}-\rho _{32}), \nonumber \\ \rho _{23}(t)= & {} \rho _{32}^*(t)= \left(\frac{3}{2}+2t\right) \rho _{11} e^{-4t}+\frac{1}{8}(-12\rho _{11}+2\rho _{22}+2\rho _{23}+2\rho _{32})e^{-4t} -\frac{1}{2}(-\rho _{22}+\rho _{33})e^{-2t}\nonumber \\{} & {} +\frac{1}{4}(-\rho _{22}-\rho _{33}+\rho _{23}+\rho _{32}), \nonumber \\ \rho _{24}(t)= & {} \rho _{42}^*(t)= (\rho _{12}-\rho _{13})(e^{-4t}-2e^{-2t})+\frac{1}{2}(6\rho _{12}-2\rho _{13}+\rho _{24}+\rho _{34})e^{-2t}+\frac{1}{2}(\rho _{24}-\rho _{34}), \nonumber \\ \rho _{34}(t)= & {} \rho _{43}^*(t)= (\rho _{12}-\rho _{13})(e^{-4t}-2e^{-2t})+\frac{1}{2}(6\rho _{12}-2\rho _{13}+\rho _{24}+\rho _{34})e^{-2t}-\frac{1}{2}(\rho _{24}-\rho _{34}), \nonumber \\ \rho _{44}(t)= & {} 1-[\rho _{11}(t)+2\rho _{22}(t)]. \end{aligned}$$

As time goes to infinity, the system approaches its steady state. So, by taking $\lim _ {t \rightarrow \infty } \rho (t)$, we arrive at

11 $$\begin{aligned} \rho (\infty )= \begin{bmatrix} 0 &{}\quad 0 &{}\quad 0 &{}\quad 0\\ 0 &{}\quad P_1 &{}\quad -P_1 &{}\quad P_2\\ 0 &{}\quad -P_1 &{}\quad P_1 &{}\quad -P_2\\ 0 &{}\quad P_2^* &{}\quad -P^*_2 &{}\quad P_3\\ \end{bmatrix}, \end{aligned}$$

where

12 $$\begin{aligned} P_1= & {} \frac{1}{4}(\rho _{22}+\rho _{33}-\rho _{23}-\rho _{32}),\nonumber \\ P_2= & {} \frac{1}{2}(\rho _{24}-\rho _{34}),\nonumber \\ P_3= & {} 1-2P_1. \end{aligned}$$

Equations (9)–(12) imply that the system is driven toward its steady state under the action of the global environment. As can be found, this steady state solution depends on the initial state of the system, hence it would be fruitful dissipative quantum computation. Note that, the steady state of the system for a generic ${\bar{n}}$ has been presented in Appendix A.

Here, it is worth noticing to mention that the evolution of an open system is usually described by the Kraus representation^44^. Therefore, the steady state density matrix (11) is recast as the following Kraus representation

13 $$\begin{aligned} \rho (\infty )=\sum _{j=1}^{4}K_{j}\rho (0)K_{j}^\dagger , \end{aligned}$$

where the corresponding Kraus operators $K_{j}$ can be obtained as

14 $$\begin{aligned}{} & {} K_1=\frac{1}{2} \begin{bmatrix} 0 &{}\quad 0 &{}\quad 0 &{}\quad 0\\ 0 &{}\quad 1 &{}\quad -1 &{}\quad 0\\ 0 &{}\quad -1 &{}\quad 1 &{}\quad 0 \\ 0 &{}\quad 0 &{}\quad 0 &{}\quad 2\\ \end{bmatrix}, \quad K_2= \begin{bmatrix} 0 &{}\quad 0 &{}\quad 0 &{}\quad 0\\ 0 &{}\quad 0 &{}\quad 0 &{}\quad 0\\ 0 &{}\quad 0 &{}\quad 0 &{}\quad 0 \\ 1 &{}\quad 0 &{}\quad 0 &{}\quad 0\\ \end{bmatrix}, \nonumber \\{} & {} K_3= \begin{bmatrix} 0 &{}\quad 0 &{}\quad 0 &{}\quad 0\\ 0 &{}\quad 0 &{}\quad 0 &{}\quad 0\\ 0 &{}\quad 0 &{}\quad 0 &{}\quad 0 \\ 0 &{}\quad 0 &{}\quad 0 &{}\quad 0\\ \end{bmatrix}, \quad K_4=\frac{1}{\sqrt{2}} \begin{bmatrix} 0 &{}\quad 0 &{}\quad 0 &{}\quad 0\\ 0 &{}\quad 0 &{}\quad 0 &{}\quad 0\\ 0 &{}\quad 0 &{}\quad 0 &{}\quad 0 \\ 0 &{}\quad 1 &{}\quad 1 &{}\quad 0\\ \end{bmatrix}. \end{aligned}$$

**Figure 2:**
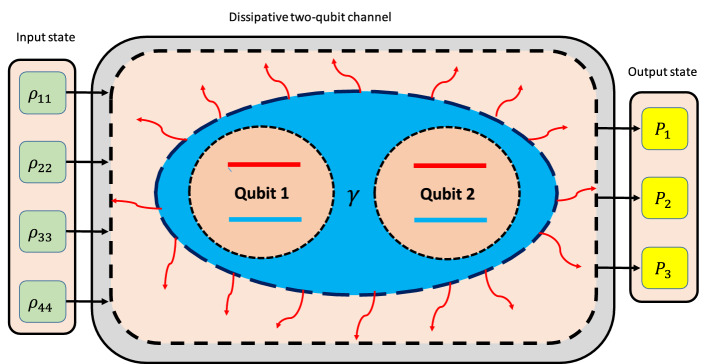
Schematic of the DQC. The channel constitutes a two-qubit system plunged into a global thermal reservoir. Since the steady state of the DQC depends on its initial state, hence it can be used for quantum machine learning. Note that, the input data are encoded into the initial density matrix of the two-qubit system. The channel maps the input data onto the steady state of the two-qubit system via the transformation, $\rho (0) \longrightarrow \rho (\infty )$.

Such transformations are frequently used in quantum computation contexts. Also, it is possible to design a quantum circuit to perform such transformations^3^. Figure 2 schematically shows how the DQC can be exploited for machine learning tasks.

As can be found from (13), the DQC acts as a quantum map that transforms the initial state of the system into its steady state. In this work, we use this feature for quantum machine learning. Indeed, we encode the input signals of the NN into the initial state of the two-qubit channel. The input signals are processed by the DQC, i.e., it can provide a superposition of the input signals. Besides, the initial state (input signals) becomes entangled due to the action of the dissipative two-qubit channel. Both properties i.e., quantum superposition and entanglement are necessary for quantum machine learning.

Now, let us find the stationary solution of the master Eq. (7) by considering a general parameterized initial state for the two qubits as

15 $$\begin{aligned} \rho _{\textrm{in}}=\rho (0)=\frac{1}{4}\left( I\otimes I+ {\varvec{r}}\cdot \varvec{\sigma }\otimes I + I\otimes {\varvec{s}}\cdot \varvec{\sigma } +\sum _{m,n=1}^3 t_{m,n} \sigma _m\otimes \sigma _n \right) , \end{aligned}$$

where ${\varvec{r}},{\varvec{s}}\in {\mathbb {R}}^3$ such that $\Vert {\varvec{r}}\Vert \le 1$, $\Vert {\varvec{s}}\Vert \le 1$, and $\varvec{\sigma }=(\sigma _1,\sigma _2,\sigma _3)$ is the vector of Pauli operators. Furthermore, ${\mathcal {T}}$ is a $3\times 3$ real matrix where its element denote by $t_{m,n}$. In general, this state is described by 15 real parameters.

It can be shown that the steady state of this dissipative two-qubit system is not unique and depends on its initial state. The stationary solution can be rewritten in terms of the two-qubit parameters (the details can be found in Appendix A). For example, for ${\bar{n}}=1$ we arrive at

16 $$\begin{aligned} \rho _{\textrm{out}}=\rho (\infty )= \begin{bmatrix} Q_1 &{}\quad 0 &{}\quad 0 &{}\quad 0\\ 0 &{}\quad Q_2 &{}\quad Q_3 &{}\quad 0\\ 0 &{}\quad Q_3 &{}\quad Q_2 &{}\quad 0\\ 0 &{}\quad 0 &{}\quad 0 &{}\quad Q_4\\ \end{bmatrix}, \end{aligned}$$

where the following quantities are obtained by the solution of the master equation at steady state regime

17 $$\begin{aligned} Q_1= & {} \frac{1}{28}(3+t_{11}+t_{22}+t_{33}), \nonumber \\ Q_2= & {} \frac{1}{56}[13-5(t_{11}+t_{22}+t_{33})], \nonumber \\ Q_3= & {} \frac{1}{56}[-1+9(t_{11}+t_{22}+t_{33})],\nonumber \\ Q_4= & {} \frac{1}{7}(3+t_{11}+t_{22}+t_{33}), \end{aligned}$$

with $Q_1+2Q_2+Q_4=1$ due to the unit trace constraint on the density matrix. The presence of off-diagonal elements in this density matrix is the signature of a quantum mechanical superposition^45^. As can be found, the steady state density matrix of the two-qubit has three independent stationary states due to the fact that $Q_4=1-Q_1-2Q_2$.

Taking a deep look at Eq. (17) shows that one arrive at these expressions $Q_2=0.5(1-5Q1)$, $Q_2=0.5(-1+9Q_1)$ and $Q_4=4Q_1$. Therefore, the general steady state of a two-qubit system interacting with a global environment can be expressed by the following quantity

18 $$\begin{aligned} q=B+A(t_{11}+t_{22}+t_{33}), \end{aligned}$$

where *A* and *B* can be controlled by the number of thermal excitations ${\bar{n}}$. Also, $t_{ii}$ ($i=1,2,3$) are the free parameters corresponding to the initial state of the two-qubit that can be used to encode quantum information. It is worth noting that the above relation holds for a generic number of thermal excitation ${\bar{n}} \ne 0$, i.e., non-zero temperatures. Hence, it may facile the implementation of quantum computation at cryogenic temperatures^46^.

## Dissipative quantum neural network

**Figure 3:**
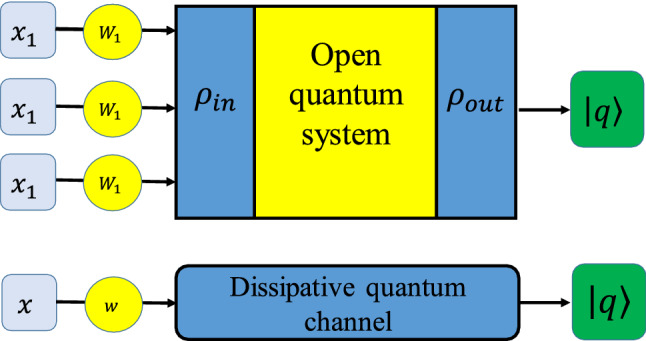
A quantum single-neuron with three input signals composed of three electro-optical modulators, a DQC, and an output signal. The initial signals are adjusted by modulators and then enter the DQC to be processed. The input and output of DQC are the initial and steady state of a two-qubit system. The output of the quantum neuron can be obtained by measuring the outcome of the DQC. The bottom part shows a compact schematic of the single neuron which constitutes the NN.

Dissipative quantum computing is established based on the theory of open quantum systems, i.e., the systems interacting with a large environment^5^. Although the total system, including a principal system plus its environment, still undergoes the unitary evolution, however, the principal system alone propagates nonunitary and experiences environmental effects. Dissipative quantum computing is of fundamental interest in QNN context because it provides new quantum computing algorithms based on dynamic attractors and steady states. Here, we want to analyze our hybrid NN model and the recipe for the construction of a quantum perceptron via a DQC. In this line, we start with a single-neuron (Fig. 3) as a building block of a NN.

Already, we showed that the steady state of a two-qubit system plunged in a thermal reservoir has three free parameters ($t_{11}$,$t_{22}$ and $t_{33}$). Accordingly, we consider a single-neuron with three input signals. At first, the signals enter an electro-optical modulator to be adjusted. Then, they pass through the DQC, so that the adjusted signals constitute the free parameters of the dissipative two-qubits system ($\rho _{\textrm{in}}$). Using Eq. (18), i.e., the steady state of the two-qubit ($\rho _{\textrm{out}}$), the output signal of this single-neuron reads as

19 $$\begin{aligned} \vert q\rangle = \left\vert b+\sum _{i=1}^{3}w_ix_i \right\rangle , \end{aligned}$$

which is the weighted sum of the neuron, where *b* and $w_i$ denote the bias and weight of the *i*-th input signal. Note that the bias and weights can be adjusted by controlling the number of thermal excitations in the environment and also via an electro-optical modulator. The above expression recalls the weighted sum of a traditional NN, however, it should be noted that the output state of a DQC is a combination of the initial states of a two-qubit system that possesses some non-classical features such as superposition and entanglement.

### Pooling layer

**Figure 4:**
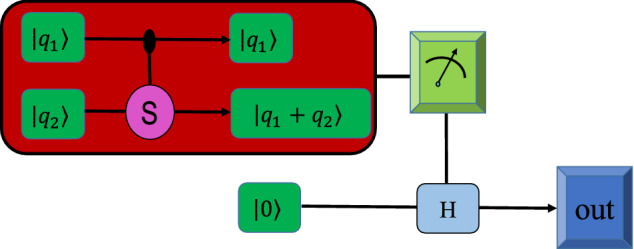
The pooling layer constitutes a SUM gate (${\mathcal {S}}$) analogous to the C-NOT gates for qubits but without the cyclic condition. The sum gate is a canonical two-mode gate for universal quantum computation based on continuous-variable quantum states. The continuous-variable gate can be realized with the quantum nondemolition technique^36^. The outcome can be obtained by the homodyne measurement.

The pooling layer can apply some quantum gates to a multi-mode quantum state and traces out unwanted modes to provide state reduction and also entanglement. At first, assume a NN composed of only two single-neurons as shown in Fig. 4. The pooling layer applies a SUM gate to the steady states of two DQCs and traces out one of them. The action of the pooling layer can be modeled by the following unitary operator^47^

20 $$\begin{aligned} {\mathcal {S}} (2)\vert q_1,q_2\rangle = \vert q_1,q_1+q_2\rangle . \end{aligned}$$

Therefore, the concatenated actions of all sum operations may be expresssed as

21 $$\begin{aligned} {\mathcal {S}}(n) \vert q_1,q_2,\cdots ,q_n\rangle = \left\vert q_1,q_1+q_2,q_1+q_2+q_3,\cdots ,\sum _{j=1}^{n}q_j \right\rangle . \end{aligned}$$

Also, we can consider the bias *b* of the NN by the action of a displacement operator on the *N*-th quantum mode which results in $b+\sum _{j=1}^{n}q_j$ as the output of the circuit. Therefore, the weighted sum corresponding to the circuit shown in Fig. 1 can be written as

22 $$\begin{aligned} \vert Q\rangle = \left\vert b+\sum _{i=1}^{n}q_i \right\rangle = \left\vert b+\sum _{i=1}^{3n}w_ix_i \right\rangle , \end{aligned}$$

where *n* denotes the number of two-qubits exploited as single-neurons. Note that the unwanted modes can be removed by tracing out them or via measurement process, i.e., $\textrm{Tr}_1(\vert q_1,q_1+q_2\rangle \langle q_1,q_1+q_2\vert )=\vert q_1+q_2\rangle \langle q_1+q_2\vert$. Here, we encode the outcome of the NN on the steady state of the last DQC. Also, the outcome of the circuit (network) can be obtained by the homodyne measurement.

### Nonlinear transformation

Modeling of dissipative QNN confronts a fundamental challenge that appears in the integration of the nonlinear, dissipative dynamics of NNs and the linear, unitary nature of quantum theory. The problem becomes more intangible if we have a look at the perceptron setup. In a NN, the incoming signals of one neuron get mapped to the output by a nonlinear function such as a step function or a sigmoid function. Such a nonlinearity results in the attractor-like dynamics that would need to contain at least two stable states that are obtained through dynamics highly dependent on the initial conditions. In the realm of quantum mechanics, we encounter the firing probabilities instead of deterministic values. Nevertheless, mapping firing probabilities onto one another by a nonlinear transformation contradicts the basic principle of the linear evolution in quantum theory^1^. The basic idea of CNNs can be generalized into the quantum domain, by replacing the McCulloch-Pitts neuron $x=\{-1,1\}$ with a quron $\vert x\rangle$ of the two-dimensional Hilbert space ${\mathcal {H}}^2$ with basis $\vert 0\rangle$ and $\vert 1\rangle$. Accordingly, the state $\vert \Psi \rangle$ of a network with *N* qurons can be introduced by a multiparticle quantum state of a $2^N$-dimensional Hilbert space ${\mathcal {H}}^{2^N}={\mathcal {H}}^2 \otimes {\mathcal {H}}^2 \otimes \cdots \otimes {\mathcal {H}}^2$ with basis $\{\vert 0,0,\cdots ,0\rangle _1,\cdots ,\vert 1,1,\cdots ,1\rangle _N\}$ may be written as

23 $$\begin{aligned} \vert \Psi \rangle =\sum _{i=1}^{2^N} a_i \vert x_1,x_2,\cdots ,x_N\rangle , \end{aligned}$$

where $a_i$, $i \in \{1,2, \cdots , 2^N\}$, denote to the complex amplitudes assigned to the respective network basis states. Zak and Williams tried to establish a quantum formalism that captures the two main properties of Hopfield networks, dissipation and nonlinearity, by replacing the step- or sigmoid-activation function with a quantum measurement^48^. They did not consider single neurons as quantum objects, but proposed a unitary walk between the quantum network basis states $\{\vert 0,0,\cdots ,0\rangle _1,\cdots ,\vert 1,1,\cdots ,1\rangle _N\}$. The evolution of the network introduced by Eq. (23) can be characterized by the following unitary transformation

24 $$\begin{aligned} b= U a, \quad a=\{a_1,\ldots , a_{2^N}\}, \quad b=\{b_1,\ldots , b_{2^N}\}. \end{aligned}$$

This unitary transformation followed by a projective measurement may result in the collapse of the superposition (23) onto the *i*-th network basis state

25 $$\begin{aligned} \{a_1,\ldots ,a_i, \ldots , a_{2^N}\} \longrightarrow \{0_1,\ldots ,1_i,\ldots , 0_{2^N}\}, \end{aligned}$$

with probability $|a_i|^2$. This map is a nonlinear, dissipative and irreversible transformation, and it can play the role of a natural quantum sigmoid function^48^. Indeed, the measurement process is an apparent exception, which can be understood as a probabilistic step-function. For instance a measurement collapses the superposition of a quron state ($\vert \mathrm quron\rangle = \alpha \vert 0\rangle + \beta \vert 1\rangle$) onto one of the basis vectors $\{\vert 0\rangle , \vert 1\rangle \}$ with probability $|\alpha |^2$ and $|\beta |^2$, respectively. The establishment of the activation process via quantum measurement has extensively been performed^48,49^. It seems to be an elegant approach for unifying the diverse dynamics of NNs and quantum theory. In analogy with the nonlinear activation process based on projective measurement, we can obtain the outcome of the DQCs by measuring their steady states with respect to the basis vectors of two-qubit system $\{\vert 1\rangle , \vert 2\rangle , \vert 3\rangle , \vert 4\rangle \}$ or any superposition of them, namely, Bell states as $\{ \vert 1'\rangle := \frac{1}{\sqrt{2}}(\vert 1\rangle + \vert 4\rangle ); \vert 2'\rangle := \frac{1}{\sqrt{2}}(\vert 1\rangle - \vert 4\rangle ); \vert 3'\rangle := \frac{1}{\sqrt{2}}(\vert 2\rangle + \vert 3\rangle ); \vert 4'\rangle := \frac{1}{\sqrt{2}}(\vert 2\rangle - \vert 3\rangle )\}$.

Superposition encapsulates the idea that the system is in all its possible states simultaneously. Only when a measurement is performed, the system collapses in one of the candidate states. Quantum measurements may be performed by a collection $\{M_m\}$ of measurement operators^3^. These operators act on the state space of the system being measured, where the index *m* refers to the measurement outcomes that may occur in the experiment. In our case, the state of the system, immediately before the measurement, is $\rho (\infty )$, then the probability that outcome *m* occurs can be obtained as

26 $$\begin{aligned} P(m)=\textrm{Tr} (\rho (\infty ) M_m^\dagger M_m), \end{aligned}$$

where $M_m=\vert m\rangle \langle m\vert$ such that $\sum _{m} M_m^\dagger M_m=\hat{{\mathbb {I}}}$. Considering the steady state of the DQC of the two-qubit, i.e., based on Eqs. (16)–(18) and using the two-qubit basis, one can easily find that the measurement outcomes reads as

27 $$\begin{aligned} P(m)= {\langle m|\rho (\infty )|m\rangle }=q, \qquad m=1,\cdots ,4, \end{aligned}$$

which is equal to Eq. (18). The above relation (27) implies that such a DQC is robust in the sense that it drives the two-qubit system towards a class of steady states with a unique form that depends on the initial state of the system. Moreover, the steady states can be controlled by adjusting the number of thermal excitations (via changing the temperature of the global environment).

Here, it should be mentioned that unitary dynamics imply that the evolution of quantum states is described by unitary operators. Physically, this means that there is no source of dissipation. This is important because inherently quantum phenomena are required for quantum computation, e.g., entanglement and quantum superposition are only maintained for a long period in the absence of dissipation or other kinds of noise. Usually, such environmental effects and thermal noises wash away these quantum effects. The idea to avoid as much as possible interactions with the environment was dominant for a long time. Nowadays, the usefulness of the environment has been put forward and also demonstrated experimentally. For instance, if a system is exposed to the thermal noise, one expects that it goes into random states. In contrast, we show that it is possible to manipulate a two-qubit system towards some certain steady states in the presence of thermal noise. Such controllable states are the key constituents of a realistic quantum computation model.

### Universal quantum computation

To design a real quantum computer, one must know how to decompose any valid quantum computation protocol into a sequence of elementary single and double quantum gates that can be exploited in physical systems. Such a set of quantum gates is required to be universal, i.e., capable of performing any valid quantum operation for a general computational task. Traditionally, the set of all single-qubit quantum gates in conjunction with a two-qubit gate called C-NOT constitutes a universal gate set. So far, several equally good universal gate sets have been proposed for universal quantum computation. The well-known operations, i.e., rotations, displacements, squeezing and beam-splitter transformations, combined with any nonlinear gate constitute a universal gate set^24^. Meanwhile, a two-qubit operation that exchanges the interaction between two qubits is as powerful as the C-NOT gate as far as computational universality is concerned. Such an operation may be realized in realistic two-qubit systems with the help of the dissipation process. Above, we showed that a DQC can be thought of as a two-qubit gate due to the fact that the global environment connects two qubits in the absence of any direct interaction between them and provides quantum superposition and entanglement even in the steady state regime. Moreover, the outcome of the DQCs may be concatenated via the action of sum gates as described in the previous section. In conclusion, the proposed hybrid model established based on the DQCs and sum gates facilitates the implementation of dissipative universal quantum computation.

## Hybrid classical-quantum neural network as a classifier

In this section, we intend to use the considered circuit as a classifier. First of all, we need to prepare the initial signals of the circuit by encoding the classical data into an *N*-dimensional state vector

28 $$\begin{aligned} \textbf{x} \rightarrow \vert \textbf{x}\rangle = \vert x_1\rangle \otimes \vert x_2\rangle \cdots \otimes \vert x_N\rangle , \end{aligned}$$

which may be performed by the action of a displacement operator on the the vacuum state, i.e., ${\mathcal {D}}(\textbf{x})\vert \textbf{0}\rangle =\vert \textbf{x}\rangle$. Then, the signals can be adjusted using an *N*-port linear optical interferometer as $\hat{{\mathcal {U}}} \vert \textbf{x}\rangle =\vert \textbf{w x}\rangle$, where $\textbf{w}$ is an orthogonal matrix corresponding to the action of linear interferometers on their entries^24^. These adjusted signals feed the DQCs, i.e. they constitute the initial states of two-qubits. The DQCs map them onto the steady state of two-qubit systems and the pooling layer concatenates the output signals of all DQCs. The resulted state can be obtained as $\vert w_1x_1,w_1x_1+w_2x_2,\ldots ,\sum _{i=1}^{N}{ w_i x_i}\rangle$. Then, another displacement operator can add a general bias $\textbf{b}=\{b_i\}_{i=1}^N$, $b_i \in {\mathbb {R}}$. After tracing out the unwanted modes, the output of circuit encoded in the last mode reads as $\vert \sum _{i=1}^{N}{ w_i x_i+b_N}\rangle$. Note that the bias can be considered only for the last mode of the output state wherein the outcome of the circuit is encoded. Putting these ingredients together, one arrives at the following affine transformation

29 $$\begin{aligned} \vert \textbf{x}\rangle \rightarrow \left\vert \sum _{i=1}^{N}{ w_i x_i+b_N} \right\rangle = \left\vert b+\sum _{i=1}^{N}{ w_i x_i} \right\rangle , \end{aligned}$$

in analogy with the traditional NNs. It is interesting to remark that a CNN can be embedded into quantum formalism. In fact, such a hybrid quantum-classical model can be used to run the traditional NNs, if the former does not generate nonclassical characteristics. The key difference between our hybrid classical-quantum model and the traditional model is that the DQCs provide superposition and entanglement among their entries, i.e., the steady state of each DQC is an entangled state composed of a superposition of the initial states of a two-qubit system.

Also, a nonlinear transformation is required to complete the NN model. Such a nonlinearity is introduced as $\hat{\mathbf{\Phi }}\vert \textbf{x}\rangle =\vert \phi (\textbf{x})\rangle$ where $\phi : {\mathbb {R}} \rightarrow {\mathbb {R}}$ is some nonlinear function. Consequently, by applying a sequence of quantum operations described above, one arrives at the following transformation

30 $$\begin{aligned} \vert \textbf{x}\rangle \rightarrow \left\vert \phi \left(b+\sum _{i=1}^{N}{ w_i x_i} \right) \right\rangle , \end{aligned}$$

which recalls a single-layer perceptron. Since the steady state of each DQC has three free parameters, therefore, the total number of data encoded into the initial states of *n* single-neurons is $N=3n$.

Finally, it should be noted that training such a variational QNN can be done using two different approaches: through classical simulation or directly on quantum hardware. The classical simulation consists of evaluating the cost (loss) functions and the gradients with respect to the parameters of each layer. However, by growing the size of the network, the training process becomes less tractable. Hence, the direct evaluation on quantum hardware will likely be necessary for large-scale networks.

Python libraries provide useful tools for training hybrid quantum-classical machine learning models, using both simulators and real-world quantum hardware. In particular, the logistic regression algorithm can be exploited to train the model and find the optimized parameters of the NN such as weights and bias.

Now, we proceed to solve some binary classification problems with our proposed model. Logistic regression can be utilized to deal with supervised NN and classification tasks with discrete possible results. It is a generalization of the linear regression, with adjustment to the classification tasks. In both regression methods, one should calculate the weighted sum of the input features variables and a bias. The difference between these two models is that in the former, this weighted sum is also the output of the model (i.e., the result of homodyne measurement), while in the latter the weighted sum subjects to the logistic (sigmoid) function as follows

31 $$\begin{aligned} \sigma {(\mathbf{w^T.x})}= \frac{1}{1+e^{-(w_0x_0+w_1 x_1+\cdots +w_Nx_N)}}, \end{aligned}$$

where we have set $w_0=b$. Indeed, one can consider the bias of the network as an additional input equal to unity, i.e., $x_0=1$. By considering a proper decision threshold, we can predict the outcome of the model. For instance, the outcome will be $y=1$ if $\sigma {\mathbf{(w^T.x)}}\ge 0.5$, otherwise the outcome will be $y=0$. In some cases, a different decision threshold may be warranted. In order to make the circuit learn the structure of our input data, it is necessary to define a cost (loss) function which quantifies the difference between the expected output and the predicted one. To do so, we use the following cost function

32 $$\begin{aligned} J({w})=-\frac{1}{m} \sum _{i=1}^{m} \left( y^{(i)} \right) \log \left( \sigma _{w} \left( x^{(i)} \right) \right) + \left( 1-y^{(i)} \right) \log \left( 1-\sigma _{w} \left( x^{(i)}\right) \right) . \end{aligned}$$

**Figure 5:**
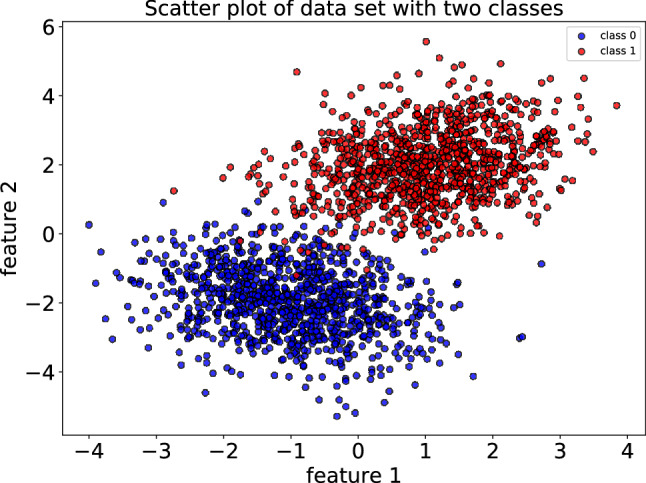
The scatter plot of data set with two classes. The data points are generated with two Guassian distributions possess different mean values and covariance matrices.

This cost function provides high probabilities for positive outcomes $(y_i=1)$ and low probabilities for negative outcomes $(y_i=0)$. The set of circuit parameters is updated at each training iteration in order to approach the minimum of the cost function. Also, the final choice of circuit parameters (updated weights) provides a set of labels (or probabilities) which is hopefully as close as possible to the real ones. The details of training the logistic regression model can be found in Appendix B. Here, we focus on solving the classification problems. To do so, we are interested to utilize our hybrid NN model as a classical-quantum classifier to solve some classification problems. Indeed, a CNN is trained by a classical computer to obtain the optimized weights and bias corresponding to the classification problem. In practice, the free parameters of the variational quantum circuit are obtained by training the proposed hybrid classical-quantum model using the logistic regression method. At first, we consider a binary classification problem with two features. Using two-dimensional normal distribution, we generate two synthetic data sets referred to as class 0 and class 1 introduced by the following features

33 $$\begin{aligned} \mu _1= & {} [-1,-2], \quad \Sigma _1= \begin{bmatrix} 1 &{}\quad -0.25\\ -0.25 &{}\quad 1\\ \end{bmatrix}, \nonumber \\ \mu _2= & {} [1,2], \quad \Sigma _2= \begin{bmatrix} 1 &{}\quad 0.25\\ 0.25 &{}\quad 1\\ \end{bmatrix}, \end{aligned}$$

where $\mu _k$ and $\Sigma _k$ denote the mean vector and covariance matrix of the *k*-th class. We consider 1000 sample points for each class and train the model with $75\%$ of the total data, while the remaining are assigned to the test set. The scatter plot of the data sets with two classes is shown in Fig. 5.

**Figure 6:**
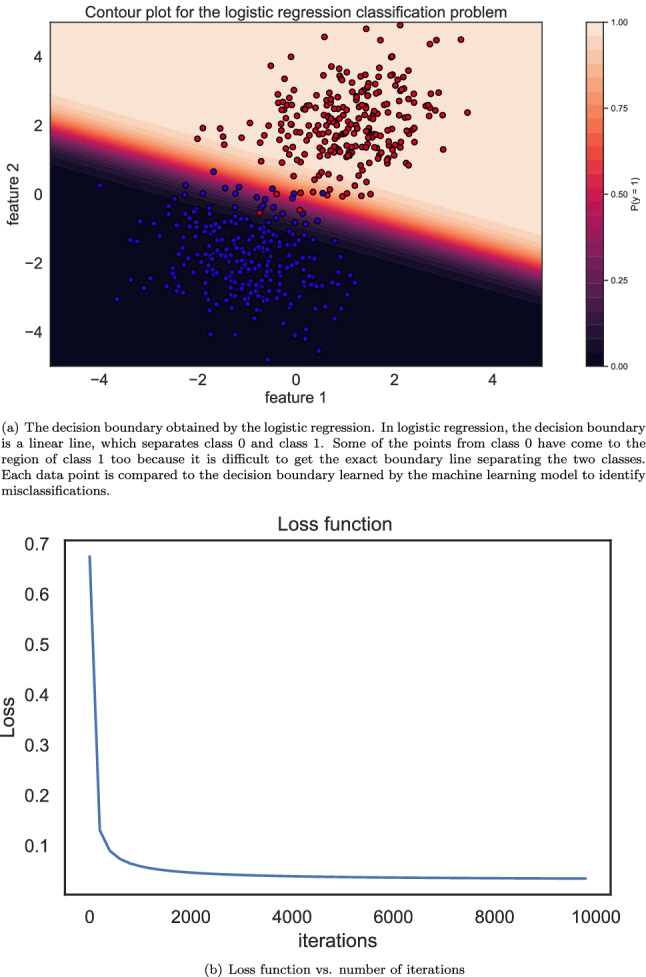
The results of the logistic regression for this binary classification problem. As can be found most of the points are correctly classified, i.e., only 7 out of 500 points corresponding to the test dataset are misclassified. The loss function demonstrates that the convergence may be achieved after 2000 iterations.

The results of classification on the test data set containing $25\%$ of the samples and the corresponding loss function obtained using the logistic regression approach are presented in Fig. 6. As can be found, most of the points are correctly classified, i.e., only 7 out of 500 points corresponding to the test dataset are misclassified. The misclassified data points are somehow located either near the boundary between both classes or in the area of the opposite class. Also, the loss function implies that about 2000 iterations are required for convergence to the solution.

**Figure 7:**
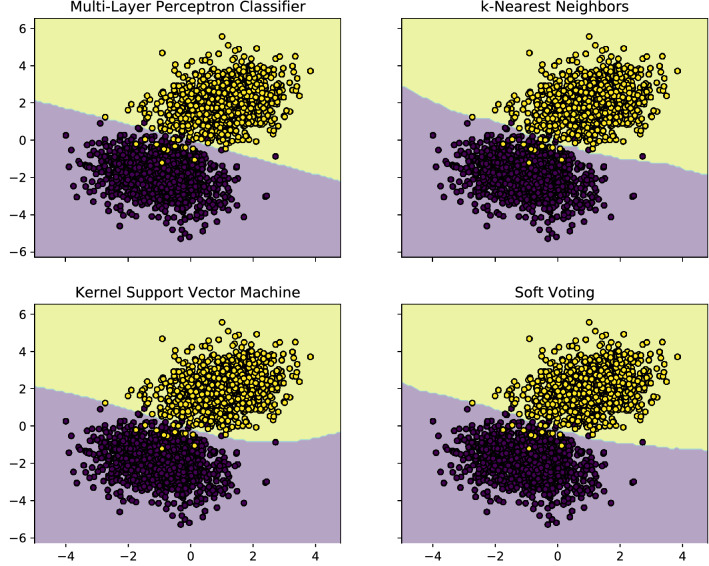
The results of different classification algorithms correspond to the whole two-dimensional synthetic dataset. All classification algorithms present almost the same accuracy for this special binary classification problem. Although there is no considerable difference between the results corresponding to this problem based on our hybrid classical-quantum model presented in Fig. 6 and the results of classical algorithms shown in this figure, the key point is that our proposed model exploits quantum resources, i.e., entanglement and superposition for dealing with machine learning tasks. Hence, it can perform computation significantly faster than the best classical algorithms.

Also, we compare the result of our proposed model with the well-known classical machine learning algorithms such as Multi-layer Perceptron, *k*-Nearest Neighbors, kernel Support Vector Machine (SVM), and Soft Voting classifiers. Here, we briefly introduce these machine learning algorithms whose details can be found in relevant literature^50–53^. Multi-layer Perceptron classifier optimizes the log-loss function using the Broyden-Fletcher-Goldfarb-Shanno algorithm or stochastic gradient descent approach. It is a popular algorithm for parameter estimation in machine learning^50^. The *k*-Nearest Neighbors algorithm is a non-parametric supervised learning method that can be used for dealing with classification and regression problems. In this case, the input consists of the *k* closest training examples in a data set. An object is classified by a plurality vote of its neighbors where the object being assigned to the class most common among its *k* nearest neighbors (*k* is a positive integer)^51^. The kernel SVM is another type of supervised machine learning algorithm. The kernel function takes data as input and transforms it into the required form of processing data. The SVM classifiers are capable of performing binary and multi-class classification problems, especially useful with high dimensional data sets^52^. Generally, a voting classifier is an estimator that combines models representing different classification algorithms associated with individual weights for confidence. The voting classifier comes with multiple voting options such as hard and soft voting options^53^. It is noticeable that, all these classification algorithms can be found in Python libraries for machine learning. Also, it is possible to generalize them for desired machine learning tasks. The contour plots corresponding to these classifiers are shown in Fig. 7. As can be found there is no considerable difference between the results of our hybrid classical-quantum classifier with the best classification algorithms. However, the decision boundaries, the lines which separate the two classes, corresponding to our proposed model and Multi-layer Perceptron classifier are linear lines because both of them are established based on logistic regression, while other classifiers possess non-linear (curved) decision boundaries. Again, we would like to emphasize that our model is established based on the advantages of superposition-based quantum computing and parallel-processed neural computing at the same time. It should be emphasized that, a quantum algorithm can just be performed on a quantum computer. Also, in principle, it is possible to run all classical algorithms on the quantum computers. However, the term quantum algorithm is applied to those algorithms that exploit at least one of the quantum features, i.e., superposition or entanglement, to perform a calculation. These quantum features result in some key differences between the performance of classical and quantum computers. In particular, entanglement as a key quantum resource allows us to process and store exponentially more information than a classical computer^3,54^. Hence, the hybrid classical-quantum machine learning algorithms based on DQC may provide the potential to revolutionize computing for certain classes of problems that cannot be solved by classical machine learning algorithms. The advantage of quantum algorithms is that, they can solve some problems significantly faster than classical algorithms^55–57^.

**Figure 8:**
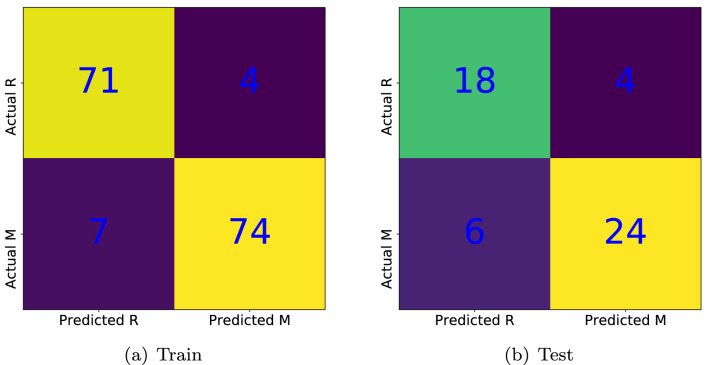
The confusion matrices correspond to the sonar dataset. The accuracies of logistic regression for both train and test datasets are about 0.93 and 0.82, respectively. Note that 187 out of 208 patterns are correctly classified which implies a good classifier with an overall accuracy of about $90 \%$.

As the second example, we want to solve a more realistic classification problem, i.e., we deal with the well-known Sonar dataset. The task is to train our variational hybrid network to discriminate between sonar signals bounced off a metal cylinder and those bounced off a roughly cylindrical rock. The dataset contains 208 patterns where 111 patterns correspond to metal cylinders and the remaining patterns come from rock cylinders at various angles and under various conditions. Each pattern is characterized by 60 numbers in the range of 0.0 to 1.0. The label associated with each record contains the letter “R” if the object is a rock and “M” if it is a mine (metal cylinder). Indeed, we encounter a binary classification wherein the patterns are classified into two classes, i.e., rock and mine. As in the case of the first example, we divide the dataset into two parts for the training and test process where $75\%$ of the total patterns are assigned to train the model and optimize the free parameters of the NN via the logistic regression algorithm. The confusion matrices corresponding to the training and test datasets are shown in Fig. 8. The detailed numerical results can be found in Tables 1, 2.

**Table 1:** The classifier’s performance is obtained based on the training data corresponding to the imbalanced dataset.

Class	Precision	Recall	f1-score	Support
Rock	0.95	0.91	0.93	78
Mine	0.91	0.95	0.93	78
Total/Ave	0.93	0.93	0.93	156

**Table 2:** The network’s performance is obtained based on the test data corresponding to the imbalanced dataset.

Class	Precision	Recall	f1-score	Support
Rock	0.82	0.75	0.78	24
Mine	0.80	0.86	0.83	28
Total/Ave	0.81	0.81	0.81	52

Not surprisingly, the network’s performance depends on the chosen dataset and the sizes of training and test samples. In order to survey the performance of our model, we redo the training process and split the data into two equal parts so that both training and test datasets possess 104 patterns. In this case, the network’s accuracy corresponding to the training and test are 0.97 and 0.78, respectively. The network’s accuracy on the training (test) set increases (decreases) somewhat with respect to the imbalanced dataset. The general network’s performance on the training set is better when the training and test sets possess the same number of patterns, i.e, the balanced dataset. In contrast, the numerical analysis shows that using the imbalanced dataset may improve the performance of the network on the test set. The details of numerical results corresponding to the balanced dataset can be found in Tables 3, 4.

**Table 3:** The classifier’s performance is obtained based on the training data corresponding to the balanced dataset.

Class	Precision	Recall	f1-score	Support
Rock	0.96	0.97	0.97	47
Mine	0.98	0.97	0.97	57
Total/Ave	0.97	0.97	0.97	104

**Table 4:** The network’s performance is obtained based on the test data corresponding to the balanced dataset.

Class	Precision	Recall	f1-score	Support
Rock	0.69	0.81	0.75	42
Mine	0.85	0.76	0.80	62
Total/Ave	0.79	0.78	0.78	104

## Summary and conclusion

Modeling QNN encontours a fundamental challenge appears in the integration of the nonlinear, dissipative dynamics of attractor-based NNs and linear, unitary quantum computation. Dissipative quantum computing is highly interesting for QNN researches because it promises tractable quantum algorithms based on the dynamic attractors and steady states. The paper outlined this challenge and established the requirements for a meaningful QNN. In particular, we introduced a hybrid classical-quantum for universal quantum computation based on open quantum systems that exploit both the advantages of quantum physics and parallel-processed NN computing, simultaneously. In this line, we designed a variational quantum circuit to establish a hybrid classical-quantum NN for doing supervised learning tasks. At first, we considered the master equation describing the dynamics of a two-qubit system plunged in a global environment and obtained the steady state of the system. Since the steady state of two-qubits depends on its initial states, hence, it is of particular interest for QNN modeling. We described the fact how to encode the input signals of the network in the initial state of two-qubits. Physically, dissipation derives the system towards its steady state and also provides entanglement that facilitates the implementation of universal quantum computation.

In order to implement a quantum computer, a set of universal quantum gates is required that are capable of performing any valid quantum computation protocol. We demonstrated that a sequence of displacement operators in conjunction with some DQCs can be used to perform universal quantum computing. Note that we provided such a universal gate set with the help of the dissipation process. Finally, we demonstrated the capability and adaptability of our proposed model by solving some binary classification problems and also compared its results with the best classical algorithms. Although the results show that our hybrid classical-quantum model and some classical algorithms offer almost the same accuracy in solving a particular classification problem, recall that non-classical phenomena, i.e., superposition and entanglement can lead to quantum speedup resides in the ability of quantum computers to do parallel processing, in a way never imagined by classical computers. Indeed, the hybrid classical-quantum algorithms outperform their classical counterparts due to the fact that they perform a part of a computational task using a quantum machine and the other part via a classical computer.

## Supplementary Information

Supplementary Information.

## Data Availability

The datasets generated and/or analyzed during the current study are available from the corresponding author upon reasonable request.
