# Supplementary material for: Hybrid classical-quantum machine learning based on dissipative two-qubit channels
Source: Sci Rep. 2022 Nov 28;12:20440. doi: 10.1038/s41598-022-24346-8 (PMC9705547; doi:10.1038/s41598-022-24346-8)
Supplement: Supplementary file 1 — Supplementary Information. [file 41598_2022_24346_MOESM1_ESM.pdf]

Appendix A: Steady state solution of master equation, i.e., Eq. (5)

Assume a general initial state for a two-qubit system

$$\rho(0) = \begin{bmatrix} \rho_{11} & \rho_{12} & \rho_{13} & \rho_{14} \\ \rho_{21} & \rho_{22} & \rho_{23} & \rho_{24} \\ \rho_{31} & \rho_{32} & \rho_{33} & \rho_{34} \\ \rho_{41} & \rho_{42} & \rho_{43} & \rho_{44} \end{bmatrix}, \tag{1}$$

For $\bar{n} > 0$, the solution of the master equation in the steady state regime can be obtained as

$$\rho(\infty) = \begin{bmatrix} Q_1 & 0 & 0 & 0 \\ 0 & Q_2 & Q_3 & 0 \\ 0 & Q_3 & Q_2 & 0 \\ 0 & 0 & 0 & Q_4 \end{bmatrix}, \tag{2}$$

where

$$\begin{aligned} Q_1 &= \rho_{11}(\infty) = \frac{f_{11}(\bar{n})}{g_1(\bar{n})}(\rho_{11}+\rho_{44}) + \frac{f_{12}(\bar{n})}{g_1(\bar{n})}(\rho_{22}+\rho_{33}+\rho_{23}+\rho_{32}), \\ f_{11}(\bar{n}) &= \sqrt{\bar{n}(\bar{n}+1)}(8\bar{n}^5+12\bar{n}^4+12\bar{n}^3+4\bar{n}^2)+8\bar{n}^6+16\bar{n}^5+8\bar{n}^4, \\ f_{12}(\bar{n}) &= \sqrt{\bar{n}(\bar{n}+1)}(4\bar{n}^5+6\bar{n}^4+6\bar{n}^3+2\bar{n}^2)+4\bar{n}^6+8\bar{n}^5+4\bar{n}^4, \\ g_1(\bar{n}) &= \frac{1}{4}(\sqrt{\bar{n}(\bar{n}+1)}(3\bar{n}^3+6\bar{n}^2+4\bar{n}+1)+3\bar{n}^4+3\bar{n}^3+\bar{n}^2)(\bar{n}^2+2\bar{n}+1+\bar{n}\sqrt{\bar{n}(\bar{n}+1)}), \end{aligned} \tag{3}$$

$$\begin{aligned} Q_2 &= \rho_{22}(\infty) = \frac{f_{21}(\bar{n})}{g_2(\bar{n})}(\rho_{11}+\rho_{44})) + \frac{f_{22}(\bar{n})}{g_2(\bar{n})}(\rho_{22}+\rho_{33}) + \frac{f_{23}(\bar{n})}{g_2(\bar{n})}(\rho_{23}+\rho_{32}), \\ f_{21}(\bar{n}) &= -\big[\sqrt{\bar{n}(\bar{n}+1)}(8\bar{n}^5+20\bar{n}^4+24\bar{n}^3+16\bar{n}^2+4\bar{n})+8\bar{n}^6+24\bar{n}^5+24\bar{n}^4+8\bar{n}^3\big], \\ f_{22}(\bar{n}) &= -\big[\sqrt{\bar{n}(\bar{n}+1)}(16\bar{n}^5+40\bar{n}^4+52\bar{n}^3+38\bar{n}^2+14\bar{n}+2)+16\bar{n}^6+48\bar{n}^5+52\bar{n}^4+24\bar{n}^3+4\bar{n}^2\big], \\ f_{23}(\bar{n}) &= \big[\sqrt{\bar{n}(\bar{n}+1)}(8\bar{n}^5+20\bar{n}^4+28\bar{n}^3+22\bar{n}^2+10\bar{n}+2)+8\bar{n}^6+24\bar{n}^5+28\bar{n}^4+16\bar{n}^3+4\bar{n}^2\big], \\ g_2(\bar{n}) &= -\frac{1}{8}(\bar{n}^2+2\bar{n}+1+\bar{n}\sqrt{\bar{n}(\bar{n}+1)})(\bar{n}^2+(\bar{n}+1)\sqrt{\bar{n}(\bar{n}+1)})(4\bar{n}^2+3\bar{n}), \end{aligned} \tag{4}$$

$$\begin{aligned} Q_3 &= \rho_{23}(\infty) = \frac{f_{31}(\bar{n})}{g_2(\bar{n})}(\rho_{11}+\rho_{44})) + \frac{f_{32}(\bar{n})}{g_2(\bar{n})}(\rho_{22}+\rho_{33}) + \frac{f_{33}(\bar{n})}{g_2(\bar{n})}(\rho_{23}+\rho_{32}), \\ f_{31}(\bar{n}) &= -\big[\sqrt{\bar{n}(\bar{n}+1)}(8\bar{n}^5+20\bar{n}^4+24\bar{n}^3+16\bar{n}^2+4\bar{n})+8\bar{n}^6+24\bar{n}^5+24\bar{n}^4+8\bar{n}^3\big], \\ f_{32}(\bar{n}) &= \big[\sqrt{\bar{n}(\bar{n}+1)}(8\bar{n}^5+20\bar{n}^4+28\bar{n}^3+22\bar{n}^2+10\bar{n}+2)+8\bar{n}^6+24\bar{n}^5+28\bar{n}^4+16\bar{n}^3+4\bar{n}^2\big], \\ f_{33}(\bar{n}) &= -\big[\sqrt{\bar{n}(\bar{n}+1)}(16\bar{n}^5+40\bar{n}^4+52\bar{n}^3+38\bar{n}^2+14\bar{n}+2)+16\bar{n}^6+48\bar{n}^5+52\bar{n}^4+24\bar{n}^3+4\bar{n}^2\big]. \end{aligned} \tag{5}$$

The last element of stationary solution is $Q_4 = \rho_{44}(\infty) = 1 - Q_1 - 2Q_2$.
For example, by considering $\bar{n} = 1$, the general stationary solution reduces to the following expressions

$$\begin{aligned} Q_1 &= \rho_{11}(\infty) = \frac{1}{14}(\rho_{22}+\rho_{33}+\rho_{23}+\rho_{32}) + \frac{1}{7}(\rho_{11}+\rho_{44}), \\ Q_2 &= \rho_{22}(\infty) = \frac{1}{28}(9[\rho_{22}+\rho_{33}]+5[\rho_{23}+\rho_{32}]) + \frac{1}{7}(\rho_{11}+\rho_{44}), \\ Q_3 &= \rho_{23}(\infty) = \frac{1}{28}(-5[\rho_{22}+\rho_{33}]+9[\rho_{23}+\rho_{32}]) + \frac{1}{7}(\rho_{11}+\rho_{44}), \\ Q_4 &= \rho_{44}(\infty) = \frac{2}{7}(\rho_{22}+\rho_{33}+\rho_{23}+\rho_{32}) + \frac{4}{7}(\rho_{11}+\rho_{44}). \end{aligned} \tag{6}$$

where we have $Q_1 + 2Q_2 + Q_4 = \rho_{11} + \rho_{22} + \rho_{33} + \rho_{44}$.

Appendix B: Training the logistic regression model

In order to train a NN with the logistic regression model, we define the following loss function [1]

$$J(w) = -\frac{1}{m}\sum_{i=1}^{m}(y^{(i)}\log(\sigma_w(x^{(i)})) + (1-y^{(i)})\log(1-\sigma_w(x^{(i)})), \tag{7}$$

where

$$\sigma_w(x) = \sigma(w^T.x) = \frac{1}{1+e^{-(w_0+w_1x_1+\cdots+w_Nx_N)}}. \tag{8}$$

This loss function provides high probabilities for positive outcomes ($y_i = 1$) and low probabilities for negative outcomes ($y_i = 0$). Using the gradient descent method, the partial derivative of the loss function can be obtained as

$$\frac{\partial}{\partial w_j}J(w) = \frac{1}{m}\sum_{i=1}^{m}(\sigma(w^T.x^{(i)}) - y^{(i)}))x_j^{(i)} \tag{9}$$

which is the product of the prediction error with the j-th feature value averaged over all instances. Our goal with gradient descent is to find the optimal weights. Gradient descent is a method that finds a minimum of a function by figuring out in which direction (in the space of the parameters, i.e., weights and bias) the function's slope rises the most steeply, and moves in the opposite direction [1]. Using logistic regression for a problem with a very large number of features may result in overfitting. Besides early stopping, an efficient solution to avoid overfitting is to add a penalty to the loss function [2, 3]

$$J(w) = -\frac{1}{m}\sum_{i=1}^{m}(y^{(i)}\log(\sigma_w(x^{(i)})) + (1-y^{(i)})\log(1-\sigma_w(x^{(i)})) + \frac{\lambda}{2m}\sum_{j=1}^{m}w_j^2, \tag{10}$$

which leads to an additional term in the partial derivatives formula

$$\frac{\partial}{\partial w_j}J(w) = \frac{1}{m}\sum_{i=1}^{m}(\sigma(w^T.x^{(i)}) - y^{(i)}))x_j^{(i)} + \frac{\lambda}{m}w_j. \tag{11}$$

Now the last missing step is the optimization process. An optimized model should be able to generalize well from the training data to the unseen test data. Using the loss function and its partial derivative, one can calculate the error of the logistic regression model and iteratively change the parameters until reaching a point where the model cannot improve anymore. The logistic regression algorithm from scratch in Python can be exploited to train the model and find the optimized parameters of the NN such as weights and bias.

References

[1] D. Jurafsky and J. H. Martin, preparation [cited 2020 June 1] Available from: https://web. stanford. edu/˜ jurafsky/slp3 (2018).

[2] S. Y.-C. Chen, C.-M. Huang, C.-W. Hsing, and Y.-J. Kao, Machine Learning: Science and Technology **2**, 045021 (2021).

[3] X. Ying, in *Journal of Physics: Conference Series*, Vol. 1168 (IOP Publishing, 2019) p. 022022.
